# eIMRT: a web platform for the verification and optimization of radiation treatment plans

**DOI:** 10.1120/jacmp.v10i3.2998

**Published:** 2009-07-21

**Authors:** Javier Pena, Diego M González‐Castaño, Faustino Gómez, Araceli Gago‐Arias, Francisco J. González‐Castaño, Daniel Rodríguez‐Silva, Andrés Gómez, Carlos Mouriño, Miguel Pombar, Manuel Sánchez

**Affiliations:** ^1^ Departamento de Fílsica de Partículas Facultade de Física, Universidade de Santiago de Compostela Spain; ^2^ Departamento de Enxeñería Telemática Escola Técnica Superior de Enxeñería das Telecomunicacións Universidade de Vigo Spain; ^3^ Centro de Supercomputación de Galicia Santiago de Compostela Spain; ^4^ Hospital Clínico Universitario de Santiago Santiago de Compostela Spain

**Keywords:** Monte Carlo, treatment verification, treatment optimization, convolution/superposition, web application, multiplan framework, CRT planning, IMRT planning

## Abstract

The eIMRT platform is a remote distributed computing tool that provides users with Internet access to three different services: Monte Carlo optimization of treatment plans, CRT & IMRT treatment optimization, and a database of relevant radiation treatments/clinical cases. These services are accessible through a user‐friendly and platform independent web page. Its flexible and scalable design focuses on providing the final users with services rather than a collection of software pieces. All input and output data (CT, contours, treatment plans and dose distributions) are handled using the DICOM format.

The design, implementation, and support of the verification and optimization algorithms are hidden to the user. This allows a unified, robust handling of the software and hardware that enables these computation‐intensive services. The eIMRT platform is currently hosted by the Galician Supercomputing Center (CESGA) and may be accessible upon request (there is a demo version at http://eimrt.cesga.es:8080/eIMRT2/demo; request access in http://eimrt.cesga.es/signup.html).

This paper describes all aspects of the eIMRT algorithms in depth its user interface, and its services. Due to the flexible design of the platform, it has numerous applications including the intercenter comparison of treatment planning, the quality assurance of radiation treatments, the design and implementation of new approaches to certain types of treatments, and the sharing of information on radiation treatment techniques.

In addition, the web platform and software tools developed for treatment verification and optimization have a modular design that allows the user to extend them with new algorithms. This software is not a commercial product. It is the result of the collaborative effort of different public research institutions and is planned to be distributed as an open source project. In this way, it will be available to any user; new releases will be generated with the new implemented codes or upgrades.

PACS number: *87.55.kh*

## I. INTRODUCTION

Currently, all radiation treatment technology is designed for local use within a certain department of an institution. Despite the fact that many commercially available solutions deal with all of the steps in the radiotherapy chain, little effort has been devoted to information sharing among different sites. There is also a severe lack of free, thoroughly documented open tools that allow the performance of tasks such as quality assurance of dose calculations and treatment plans. This is particularly important when implementing a new radiation treatment technique, such as intensity‐modulated radiation therapy (IMRT).

To improve the quality of their radiation treatments, medical institutions must purchase new equipment and software components. Sometimes, this is done at the cost of the loss of knowledge concerning the exact details in the treatment chain. For example, it is rather difficult to precisely determine how optimization, segmentation, and sequencing proceed in a certain planning system (TPS). The process of gaining confidence in the combination of hardware and software required to deliver a complex treatment relies heavily on a well‐established quality assurance (QA) procedure.

In the case of dose calculation and optimization, the need to thoroughly understand the planning process leads many researchers to develop custom‐made Monte Carlo verification tools or in‐house systems for treatment optimization.^(15)^ There is also a wealth of research dealing with the estimation of deviations in calculated and delivered dose, both in absolute or relative terms.^(^ ^6^ ^–^ ^8^ ^)^ However, most clinical institutions rely on locally available commercial QA tools.

Another approach to the further improvement of radiation treatment delivery is related to information sharing. RT departments willing to implement a new technique may benefit from the learning curve of another institution that has already commissioned it. However, there is a severe lack of tools for that purpose, although some incipient work exists.^(9)^

There have been valuable attempts to overcome all of these drawbacks in the form of publicly available radiotherapy‐specific Monte Carlo codes^(^ ^10^ ^–^ ^13^ ^)^ or software environments such as CERR^(14)^ and MMCTP.^(15)^ However, these tools also have certain drawbacks such as a dependence on commercial software licenses (CERR) or high‐performance local computational infrastructures (MMCTP).

On the other hand, the emergence and generalization of Internet information technologies, the availability of broadband connections, and the development of new interactive web technologies have opened up many possibilities for the radiotherapy world. The eIMRT project was designed to provide high‐end radiotherapy tools to the medical physics community through simple interfaces. This approach is in agreement with the new vision of the Internet as a service provider, in addition to its classical use as a content provider. There have been some previous experiments in remote (yet partially manual) radiotherapy^(16)^ and commercial tools will probably evolve in that direction in the near future. Nevertheless, we are not aware of the existence of any open multitier platform like ours that enables fast integration of new modules for verification or optimization, regardless of their computational requirements.

The eIMRT platform provides access to Monte Carlo verification, treatment optimization, and a dataset of interesting treatments/clinical cases through a user‐friendly, platform and browser independent web page. This approach is advantageous for the final users because the implementation and maintenance aspects of those resources are transparent. The access to the platform only requires a standard web browser. Section II (below) describes the components of the open platform and discusses the possibilities of the different tools. eIMRT is an off‐line tool because the elapsed time between requesting a certain computation service and actually obtaining the results is generally on the order of ten minutes for optimization to a few hours for verification using an inhomogeneous cluster of Intel Xeon (single, dual, and quadcore connected by GigaEthernet). However, these times can be improved when new parallelization becomes available, mainly for optimization processes. Requests are sequentially computed and, since no computing resources are reserved for each user, result availability depends on platform workload. After completing the calculations, the user is informed by e‐mail and can log in again and review the results.

In its current version, eIMRT is designed to support different users from multiple institutions. The initial scope of the eIMRT platform was to provide its services within a national area. Nevertheless, it has been tested by different European radiotherapy services. Its main aim is assisting practitioners with timely issues or suggesting alternative treatment strategies. In spite of the fact that the platform is hosted and administered by the Galician Supercomputing Center, it is a flexible architecture and may be updated, changed, and improved according to user requests and contributions. As an open source project, its open software platform will be available to any user. Additionally, it will be maintained through the collaborative effort of different developers and institutions that can provide new code for the verification and optimization functions. The new versions will be periodically consolidated and released by the project managers.

## II. MATERIALS AND METHODS

### A. Platform architecture

The platform follows a client‐server architecture.^(17)^

- The client side is a web browser supporting Macromedia flash objects and Sun Java applets, in addition to HTML. These are common web elements today and do not require specific programs. No special computer or bandwidth requirements are necessary. A low‐end computer and a typical DSL connection are enough to access the platform.
- The server side follows a two‐tier architecture: web services and computational modules. These modules may either be self‐contained or Grid‐oriented (multitier architecture). Diverse components of the platform such as treatment optimization, treatment verification, or gamma maps calculation^(18)^ are handled through web services. The web server that holds the web page also acts as the web services client and database server. Finally, a computing interface connects the computation nodes (either a local cluster or a Grid infrastructure) with the web server.

### B. Management of users and user data

The platform offers a login/password interface for user authentication. Users from the same institution may have different privileges. Institution administrator is the only user allowed to define and commission accelerators and CT data, upload, and erase treatments while other users can submit treatment verification and/or optimization jobs.

The eIMRT platform requires the user to define and commission its accelerators and tomographs prior to treatment submission and computing. Once the accelerators are identified within the list of supported machines, it is possible to commission them for each dose calculation algorithm. This process is fully automated and requires the submission of PDD and profiles measured in a water phantom for field sizes of $20\times 20\;{\text{cm}}^{2},\;10\times 10\;{\text{cm}}^{2}$, and $2\times 2\;{\text{cm}}^{2}$. For the Monte Carlo commissioning, a series of accelerator head simulations are run for different combinations of the primary beam energy and FWHM. Although results are dependent on the quality of measurements, a combined uncertainty of 0.2 MeV for the energy and 0.2 mm for FWHM are achievable.^(19)^ However, the user is requested to validate the commissioning of the accelerator before its first usage. This ensures that inaccurate machine models are not used afterwards in the calculations. The actual process of automatic commissioning depends on the dose calculation algorithm and is described in the following sections.

CT data, on the other hand, are defined by providing a label identifier and a calibration table that associates Hounsfield units to density values and materials. Both in optimizations and verifications, the user has to specify the CT that was employed to perform the patient scan, in order to select the proper ramp to generate the patient density map. The user also has to enter the DICOM files associated with the treatments (CT, plan, etc.).

The data exchange between the client and the server is handled by a secured connection (HTTPS security protocol). Nevertheless, in order to preserve the confidentiality of patient data, all DICOM files are rendered anonymous by a JAVA applet before leaving the client side. It is completely impossible to restore these data in the server or during the transport through the Internet.

### C. Monte Carlo treatment verification

Monte Carlo treatment verification consists in the comparison of the TPS‐calculated dose distribution and the dose distribution calculated by Monte Carlo for the same radiation treatment plan. The objectives of such a comparison are to detect regions of dose disagreement and to study the possible origins and consequences for the patient. This method is particularly suitable for detecting underdosages or overdosages to regions around the interface between materials of different densities.

In eIMRT, Monte Carlo simulations are performed using the BEAMnrc package.^(11)^ The current version supports unwedged photon beams. The accelerator head is simulated from the bremsstrahlung target to the bottom of a multileaf collimator employing BEAMnrc. Dose deposition inside the patient is simulated with the DOSXYZnrc code.^(20)^ In addition to the accelerator and CT commissioning files, it is necessary to provide CT data and the treatment plan in DICOM format for each patient. The calculation of figures of merit, such as the gamma map(18) for treatment verification, requires the submission of a DICOM RT dose file containing the TPS‐calculated dose distribution.

**Figure 1:**
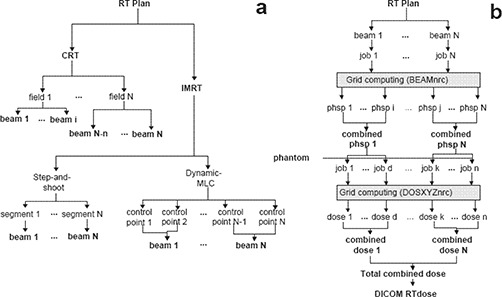
Generation of BEAMnrc simulation beams from an RT Plan file for: (a) CRT, IMRT step‐and‐shoot, and dynamic‐MLC IMRT; (b) input generation, execution and combination of Monte Carlo accelerator and patient simulations.

Every beam in the plan is decomposed into sub‐beams for simulation. In conformal treatments, only a few beams with different directions of incidence are necessary. eIMRT performs different parallel simulations associated with each beam incidence to decrease accelerator simulation time. On the other hand, IMRT treatments follow a different approach. In step‐and‐shoot IMRT plans, a simulation is created for each segment of the treatment. The number of histories associated to each segment simulation is proportional to its monitor units (MU). The radiation intensity distribution in dynamic‐MLC IMRT treatments is created by specifying the position of the leaves at certain times of the treatment. These associations of leaf positioning and treatment times are called *control points* and *N of* them define a dynamic‐MLC beam. From these *N* control points, eIMRT generates *N‐1* static beams, such that the position of the leaves in beam *i* corresponds to the average position of the leaves in control points *i* and i+1. As in an ordinary dynamic‐MLC treatment, *N* is in the order of 1000; this definition of the beams in the simulation accurately reproduces the actual intensity distribution. The subplot *a* in Fig. 1 summarizes this procedure (also valid for step‐and‐shoot and sliding window modalities).

The accuracy of a Monte Carlo simulation, provided that the simulation code has been thoroughly tested (as BEAMnrc has been), depends on two key factors: the accuracy of the accelerator commissioning,^(19)^ and the QA of the process of input generation, simulation, and collection of results.

The quality assurance of the simulations was handled by storing simulation parameters in intermediate control files and checking simulation results afterwards. The accelerator geometry is stored in a data file for each available type. After processing the treatment plan to generate the beam parameters to be simulated, the eIMRT application loads the geometry of the accelerator and builds BEAMnrc input files on‐the‐fly. This procedure of input generation allows treatments combining multiple modalities and multiple isocenters to be simulated. It also allows the calculation of variance reduction parameters on a beam‐by‐beam basis. Directive Bremsstrahlung Splitting (DBS) is employed to increase the number of particles in the phase space. The splitting radius is calculated according to beam shape and size in order to maximize particle scoring. Range rejection with ECUT=2.0MeV is also employed.

Primary electron beam parameters are set from the accelerator commissioning user file. The primary number of particles in the simulation is set according to the weight of the beam in the whole treatment (calculated from its MU) and is stored in a control file. The different accelerator simulations are uncorrelated by setting the random generator seeds at random, using machine time as seed.

After completing all the jobs in the accelerator head simulation, the individual phase spaces are combined. During this process, each individual phase space is checked to verify that the primary number of particles that generated it (stored in the file header) equals the number specified in the inputs. In this way, incorrectly finished processes are identified and can be rerun.

The phase spaces resulting from this combination – each one corresponding to a beam incidence – are simulated against the patient geometry using the DOSXYZnrc code. The conversion of phantom coordinates from the DICOM to the BEAMnrc coordinate system is also performed in this step. After completing the phantom simulations, the dose distributions are added, weighting them according to the number of monitor units associated with each phase space (also stored in control files). This procedure is necessary as DOSXYZnrc normalizes the resulting dose per primary particle. Typically, phase spaces have to be recycled about 10 times. Reusing particles does not introduce any significant bias as the procedure of generating them from target to collimator (i.e. without recycling a primary phase space) ensures a satisfactory statistical independence.

The resulting combined dose distribution can be provided either as absolute dose (obtained from the absolute dose per primary electron) or relative dose, by normalizing to unity at maximum dose or obtaining the normalization factor by comparison with the TPS‐calculated dose distribution (if provided). As a final outcome of the verification, the user can retrieve the Monte Carlo calculated dose in DICOM RT dose format. This entire process is graphically summarized in subplot *b* of Fig. 1.

### D. Optimization of treatment plans

Treatment optimization can be applied to different issues ranging from the selection of the number and direction of beam incidences, the beam fluence matrix and treatment efficiency (in terms of TCP, NTCP, EUD…), or the number of segments to be delivered. The implementation of treatment optimization in eIMRT has been restricted to the selection of beam incidence angles and intensity map optimization, taking advantage from the large remote computational resources available.

Due to the particular characteristics of the platform (low user interactivity and off‐line calculations), optimization input and output were designed as follows:

- **Data input**. In addition to patient contours in DICOM RTstruct format and patient CT, the user is requested to provide certain optimization objectives. As no interaction during optimization is allowed, the number of constraints that can be provided per organ is relatively large. Three organ dose constraints are implemented: minimum dose, mean dose, and maximum dose. Also three dose‐volume constraints can be prescribed for each organ. It is also possible to configure whether the resulting DVH curve is expected to have a dose above or below those prescribed values in the dose‐volume points. All these constraints are graphically explained in Fig. 2. Their code implementation approximately follows the description of Wu et al.^(21)^
  **Figure 2:**
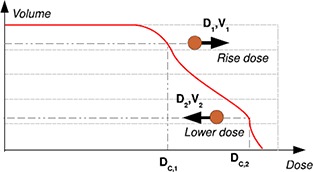
Types of dose‐volume constraints in eIMRT: dose increase $\left(\text{final}\;D_{c}>D_{1}\text{for}\;V_{1}\right)$ or dose decrease $\left(\text{final}\;D_{c,2}\&<D_{2}\text{for}\;V_{2}\right)$.
  Each organ has to be identified in terms of whether it is intended to be treated (PTV) or spared (OAR). Additionally, it is possible to select different behaviors in PTV and OAR overlapping regions. For every organ identified as an OAR, its PTV overlap can be treated either as a PTV, as an OAR, or a combination of both. It is also necessary to assign a weight to each organ for the optimization process.
- **Data output**. At the end of the optimization process the user is offered a pool of treatment plans. They can be graphically reviewed in terms of dose volume histograms (in its current version DVH is the only graphical output available). It is possible to export from the platform a DICOM‐RT file corresponding to a selected optimization for its comparison with the TPS. Afterwards, the user can change several parameters of the process (e.g. constraints and organ weights), and request a reoptimization if none of the resulting plans was entirely satisfactory. This multitreatment pool approach originates from the wide range of optimization parameters which discourages the user from selecting the best plan on the basis of the cost function only.
  eIMRT treatment optimization is evaluated by looking for the best solutions not only in terms of geometrical beam parameters (i.e. angles of beam incidence), but also in terms of treatment complexity (i.e. from conformal radiotherapy to IMRT). Consequently, the following aspects are jointly considered in the treatment optimization.
- **Number and directions of incidence of radiation beams**. The minimum and maximum number of directions of incidence (*N*) to be explored during optimization are prescribed as optimization inputs. The gantry and coach angle of each of the *N* beams of a certain solution are optimized by the platform, allowing the user to restrict the result to be coplanar (zero coach angle). The implementation of the incidence optimization strategy is described in Section II D.1 and it basically consists of a simultaneous optimization of beam orientations and beam weights.
- **Treatment strategy**. Four plan categories for different treatment strategies are provided for each beam arrangement, with a number of beams ranging between the minimum and maximum number of incidences. In addition to the CRT and IMRT plans, intermediate solutions with fewer segments than in IMRT are also provided. The implementation of these strategies in beam fluence optimization is described in Section II D. 2.

The reduction of treatment complexity is one of the advantages of providing such a large set of solutions for a certain patient. From the solutions pool, the user can evaluate the convenience of increasing or decreasing the number of beams (and thus treatment time) or the complexity of the treatment.

#### D.1. Beam incidence optimization

Beam orientation optimization has been addressed in previous works in many ways, depending on whether the treatment is conformal or intensity modulated. For conformal treatments, a typical approach is to rank each beam incidence within a test set according to its adequacy for a set of prescriptions. This strategy was employed by D'Souza et al.^(5)^ in terms of mean organ‐at‐risk (MOD) data. Rowbottom et al.^(22)^ used an exhaustive search with a priori removal of unsuitable beams.

In intensity modulation, the optimization of *N* incidences has been mainly addressed by evaluating combinations of *N* incidences selected by a heuristic algorithm, such as simulated annealing^(^ ^23^ ^–^ ^25^ ^)^ or by optimizing *N‐n* beams in some way and then adding *n* beams one by one until the final number, *N*, is achieved.

Other methods described in the literature include the direct optimization of beam incidences, including them as optimization variable s,^(26)^ or a beam‐ranking schema such as the one in CRT. ^(27)^ Early eIMRT tests have shown that the latter fails to detect the synergies among beams when they are modulated and, thus, it was discarded as an optimization strategy.

Due to the fact that the architecture of the eIMRT platform involves many non‐intercommunicating jobs, a guided exhaustive search is the best choice to optimize beam incidences. In this approach, each test solution starts by setting incidences with equally‐spaced gantry angles (even in the case of non‐coplanar treatments). Next, a certain percentage of the beams is slightly changed, choosing the incidences to be modified at random. The level of change for each of the varying incidences is chosen at random, but it is kept within half the distance between two consecutive, equally spaced beams.

In non‐coplanar treatments, the number of non‐coplanar beams and their coach angles are chosen at random. As many optimization jobs associated to the same problem use this algorithm simultaneously with the same number of beams, the testing of the same configuration in different jobs is unlikely to occur. Once the intensity map optimization is completed, the final figure of merit is compared to the results achieved by other jobs and stored in a control file. The best beam arrangement is saved on disk and presented to the user.

**Figure 3:**
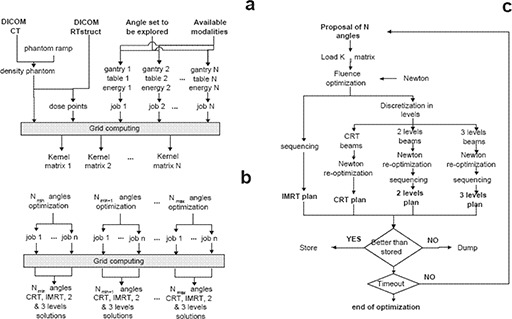
eIMRT optimization of radiotherapy treatments: (a) data pre‐process and generation of kernel matrix; (b) treatment optimization for different number of radiation beams; (c) angle and intensity matrix optimization.

For each of the incidences in the space of gantry and coach angles to be explored, a separate dose calculation is conducted, storing the associated data in separate files (subplot *a* in Fig. 3). This allows all data to be precalculated and ready for the intensity map optimization step (subplot *b* in Fig. 3). Doses are calculated by means of an algorithm developed by S.A. Naqvi et al.^(28)^

#### D.2. Intensity map optimization

eIMRT intensity or fluence map optimizations employ an implementation of the Newton gradient algorithm. Although local minima are known to exist in IMRT optimization when employing DVH constraints.^(29)^ this has been shown to have little effect on the final solution.^(30)^ Despite the fact that there is an abundant bibliography on the use of simulated annealing for IMRT optimization.^(31)^ gradient algorithms are faster in practice; in particular, those based in the Newton method.^(32)^

The contributions of the constraints to the objective function, which was defined in Section II D, can be mathematically described for a single organ *k* as quadratic functions of the dose.

Dose constraints:

Minimum dose $(D_{\min}):\;f_{d\;\min}(D)\sum_{i\in k}H(D_{\min}-D_{i})\cdot {\left(D_{i}-D_{\min}\right)}^{2}$
Mean dose $(D_{\text{mean}}):\;f_{\text{dmean}}(D)\sum_{i\in k}{\left(D_{i}-D_{\text{mean}}\right)}^{2}$
Maximum dose ${(D}_{\max}):\;f_{d\max}(D)\sum_{i\in k}H(D_{\max}-D_{i})\cdot {\left(D_{i}-D_{\max}\right)}^{2}$

DVH constraints:

Aiming to lower dose: $f_{\text{lower}}^{\text{dvh}}(D)=H(D_{c}-D_{\text{dvh}})\cdot \sum_{i\in k}H(D_{i}-D_{\text{dvh}})\cdot {\left(D_{i}-D_{\text{dvh}}\right)}^{2}$
Aiming to raise dose: $f_{\text{raise}}^{\text{dvh}}(D)=H(D_{\text{dvh}}-D_{c})\cdot \sum_{i\in k}H(D_{\text{dvh}}-D_{i})\cdot {\left(D_{i}-D_{\text{dvh}}\right)}^{2}$

In these equations, *H* represents a step function (i.e. H(x)=1for x>0 and H(x)=0for x≤0), and $D_{i}$ is the dose at a certain point *i* within organ *k*. $D_{\text{dvh}}$ is the dose value prescribed for a DVH constraint of volume $V_{\text{dvh}}$ and $D_{c}$ is the value of the dose for this same volume in the current optimization iteration.

The global objective function can be written as the sum of all constraints regarding all organs *k*, properly weighted with $w_{k}$:

(1) $$OF(D)=\frac{1}{\sum_{k}w_{k}}\cdot \sum_{k}w_{k}\cdot (f_{d\min}(D)+f_{dmean}(D)+F_{d\max}(D)+f_{lower}^{dvh}(D)+f_{raise}^{dvh}(D))$$

The value of the dose at a certain point is calculated as $D_{i}=\Sigma K_{j}\;W_{i}$ where $W_{j}$ is the intensity of beamlet *j*. This linear algorithm is based in the kernel matrix $K_{\text{ij}}$ that stands for the contribution of the fluence crossing the beamlet *j* to the dose at point *i*. In eIMRT, this kernel matrix is evaluated with Monte Carlo Kernel Superposition software (MCKS), a Monte Carlo convolution/superposition (C/S) dose calculation algorithm.^(28)^ This code transports photons individually across the phantom, sampling their interaction points and propagating the deposited energy according to in‐water precalculated kernels. The accuracy of this method is superior to other C/S dose calculation algorithms, at the cost of a higher computation time. However, the accuracy of the dose calculation during the optimization process has been shown to significantly improve the quality of radiation treatments.^(33)^

First and second partial derivatives of the quadratic terms of the objective function were estimated as in Wu and Mohan.^(21)^ The implementation of this algorithm in eIMRT allowed us to obtain an inverse IMRT solution for a certain set of beam incidences. From this optimum fluence, we realized three types of treatments result: IMRT, CRT, and pseudo‐IMRT.

- **IMRT:** The optimized fluence is sequenced for step‐and‐shoot delivery according to the method described by Xia and Verhey.^(34)^ This algorithm was chosen for the simplicity of the implementation, and it has been shown to generate few MLC segments for some treatments.^(35)^ It is possible to impose constraints on the minimum number of MU per segment and the type of MU (integer or decimal) during sequencing. The minimum segment area is set currently to 2.5 cm^2^.
- **CRT**: From the continuous fluences that are obtained by IMRT optimization, a conformal plan is also generated by calculating the average fluence (*W*) for non‐zero beamlets. All beamlets with intensity values over half this average value are set to *W*, otherwise they are set to zero. Then, the resulting intensity distribution is transformed into an intensity distribution that could be delivered by a single beam. This process implies removing blocks of zero‐valued beamlets inside the field in the first place and then calculating the collimator rotation that fits this intensity distribution. Finally, a second optimization loop employing the Newton algorithm finely tunes the intensity of each CRT field.
- **pseudo‐IMRT:** Following an analogous procedure to CRT field generation, the continuous IMRT fluence is discretized in two‐ and three‐intensity levels. This procedure is performed by grouping beamlets according to the intensity range to which they belong. Then, all beamlets within a certain range *i* are associated to their average value ${\bar{W}}_{i}$. The beamlets of the first interval whose original values were below ${\bar{W}}_{i}/2$ are cleared. After this discretization process, a second reoptimization loop finely tunes the ${\bar{W}}_{i}$ values. Lastly, each intensity level is transformed into individual segments using the same algorithm as in the full IMRT solution. However, the final plan that results from this solution is composed of rather large beams, closer to forward planning than to inverse IMRT segments. It is possible to impose cutoffs on the segment area in order to avoid tiny segments contributing to the plan.

This intensity map optimization is summarized in subplot *c* of Fig. 3.

#### D.3. Description and commissioning of accelerators in C/S dose calculation algorithms

The description of accelerators in eIMRT differs somewhat from the work of Naqvi et al.^(28)^ The radiation generated by the medical LINAC is approximated by a dual source model in which both the primary and extra‐focal sources of photons are described by a single Gaussian. The primary source on‐axis photon spectrum is modeled, as in the work of Fippel et al.,^(36)^ with a probability distribution given by Eq. (2), where TV is a normalization factor and *l* and *b* are free parameters. These last variables are related to the peak energy $\left(E_{p}\right)$ and the mean energy of the spectrum $\left(\bar{E}\right)$ as $E_{p}=l/b$ and $\bar{E}\approx (l+1)/b$ (Eq. (14) in Fippel et all.^(36)^

(2) $$p_{axis}(E)=N\cdot E^{l}\cdot e^{-bE}$$

The primary source off‐axis spectrum for a certain radius *R* at isocenter height is constructed as in Eqs. (17) and (21) in Fippel et al.:^(36)^

(3) θ=atan(R/SAD)

(4) $$s(\theta )=1/{\left(1+0.00181.\theta +0.00202.\theta^{2}+0.0000942.\theta^{3}\right)}^{1/0.45}$$

where θ is the angle with respect to beam axis expressed in degrees. The probability associated with a certain energy $E_{\theta }$ at angle θ is defined as:

(5) $$p(E_{\theta },\theta )=\frac{1}{s(\theta )}\cdot p_{axis}(E_{\theta }/s(\theta ))$$

Extra‐focal photon energies $\left(E_{ef}\right)$ are obtained^(^ ^28^ ^,^ ^36^ ^)^ by sampling a certain energy *E* from the primary on‐axis spectrum (Eq. (2)) and then applying Eq. (6) based on Compton scattering. In this last equation, φ is the angle formed by the direction vector of the photon and the vector that connects the center of the focal source with the point in the extra‐focal source plane where the photon is generated.

(6) $$E_{ef}=\frac{E}{1+(1-cos\phi )\cdot E/0.511}$$

where the photon energy *E* is expressed in MeV. The focal source fluence is constructed by sampling a modulation factor from a numerical array that defines fluence strength as a function of arc length, calculated as *SAD*·θ(*radians*), as in Naqvi et al.^(28)^ This strength is normalized to unity at zero arc length.

The commissioning of an accelerator for the MCKS algorithm in the eIMRT platform is an automated process that consists of two optimization loops. The main loop optimizes the spatial FWHM and the relative strength of the primary and extrafocal sources by means of a Levenberg‐Marquardt algorithm. The objective function of each test combination corresponds to the one‐dimensional gamma^(18)^ function of measured and calculated depth doses and lateral profiles. The second optimization loop deals with the optimization of the primary source in‐axis spectrum and the fluence modulation array for each of the proposed source sizes and intensities.

The primary in‐axis spectrum is defined by the two free parameters, *l* and *b*, of the analytical spectral distribution. The Levenberg‐Marquardt algorithm is employed to determine the values of these parameters that minimize the agreement between the simulations and measurements for the 10cm×10cm field, in terms of gamma profiles. This procedure is conducted by calculating the depth dose of monoenergetic 10cm×10cm photon beams with unity fluence modulation. The weight of each of these profiles for a certain combination of parameters *l* and *b* is given by the probability distribution (Eq. (2)). Initial test values of *l* and *b* for a certain megavoltage are taken from the linear fitting of the mean and maximum spectra values published by Sheikh‐Bagheri and Rogers.^(37)^

On the other hand, fluence modulation is optimized once the best in‐axis spectrum is available. Lateral profiles at depths higher than maximum buildup are calculated for the widest field size, assuming a constant, unity fluence modulation. The new modulation factors are obtained from the quotients of the measured and simulated lateral profiles in regions where particle equilibrium applies. These factors are employed as starting values in the simulation of the other fields, calculated in order of decreasing field size and applying the same quotient method iteratively. This procedure implements an iterative optimization of model parameters in the vicinity of the central axis.

After the in‐axis spectrum and fluence modulation are completed, one‐dimensional gamma curves between simulated and measured depth doses and lateral profiles of all field sizes are calculated. All these curves are associated to the cost function for both the primary and secondary strengths and the sizes under test.

## III. RESULTS

### A. Monte Carlo treatment verification

**Figure 4:**
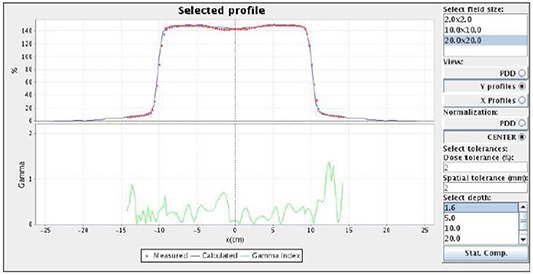
Example of commissioning of a linear accelerator in the eIMRT platform. The validation tool allows the user to compute the PDD difference and profile gamma functions between calculated and measured values.

**Figure 5:**
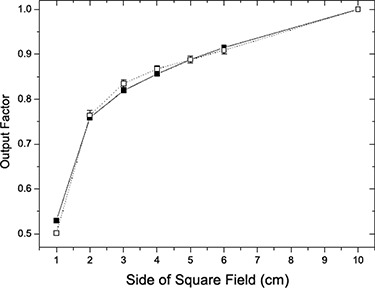
Validation of Monte Carlo commissioning of a 6MV beam. Output factors (red dots) are calculated with Monte Carlo for the selected parameters E=5.75MeV and FWHM=0.25cm, while the black squares are the quality assurance measurements performed at the clinical institution.

In the present version of the platform, only Siemens Primus and Varian Clinac 2100 are supported. Monte Carlo commissioning of 6 MV Siemens Primus accelerator from Complexo Hospitalario de Santiago showed that the comparison of measured and predicted PDDs and profiles for reference $10\times 10\;{\text{cm}}^{2}$ and $20\times 20\;{\text{cm}}^{2}$ fields (at depths of 1.5, 5, 10, 20 and 30 cm) fulfill a gamma test (with 3% and 3 mm), and can withstand the same tolerances as a commercial TPS. For gamma tolerances of 1 mm and 1% PDDs and lateral profiles, all fields (except 25cm×25cm and 30cm×30cm) were reproduced with average gammas of less than 0.7. These values were raised to a maximum average gamma of 1.4 in the lateral profiles of the 30cm×30cm field (see Fig. 4). As an offline validation of the accuracy of the commissioning procedure it is possible to compare the output factors calculated through Monte Carlo with the measurements (performed with different active volume chambers) shown in Figure 5. Maximum different in the output factors is around 2% with an average value below 1% except for the 1×1cm field.

**Figure 6:**
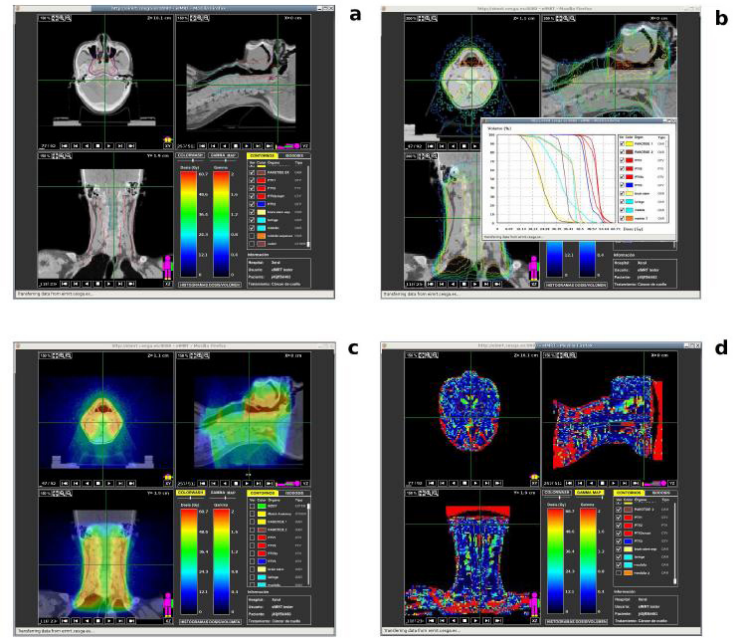
Treatment verification graphical Flash visualizer. This tool is included in the eIMRT platform to compare Monte Carlo calculated dose distribution and that provided from a TPS: (a) shows the user defined contours (PTV, OAR, etc) on the CT data image; (b) dose volume histogram and isodoses from Monte Carlo simulation; (c) color coded superposition of Monte Carlo calculated dose; (d) two‐dimensional gamma map between both dose distributions.

Figure 6 represents the outcome of a treatment verification of a head and neck tumor. The Flash visualizer employed for this purpose is the same that supports treatment files preview. It allows basic graphical navigation for slicing through the CT. The contours (plot *a* in Fig. 6) can be overimposed on the CT by selecting the organs from the tickbox.

Isodose curves can also be reviewed (plot *b* in Fig. 6) by selecting the curves corresponding to different dose percentages from another tickbox. Dose volume histogram visualization is also shown in this same plot. The tool allows the superimposition of Monte Carlo calculated dose distributions to the CT (plot *c* in Fig. 6) as well as selecting its transparency with a slidebar. Gamma maps can be visualized (plot *d* in Fig. 6) with the same procedure. In the particular case of the treatment in Fig. 6, several regions present large gamma values. This is due to a lack of particle equilibrium at both ends of the phantom during Monte Carlo simulations that is a consequence of the finite size of the CT.

The quality of the images shown by this flash tool is relative, as its purpose is to provide the user with a fast, rough approximation to the results. The clinical review of the dose distribution and the gamma map has to be performed by the user with a different tool, after retrieving the correspondent DICOM files.

The current version of our software produces gamma maps only, but the computation of any other figure of merit can be easily added as an extra software module.

### B. Treatment optimization

**Figure 7:**
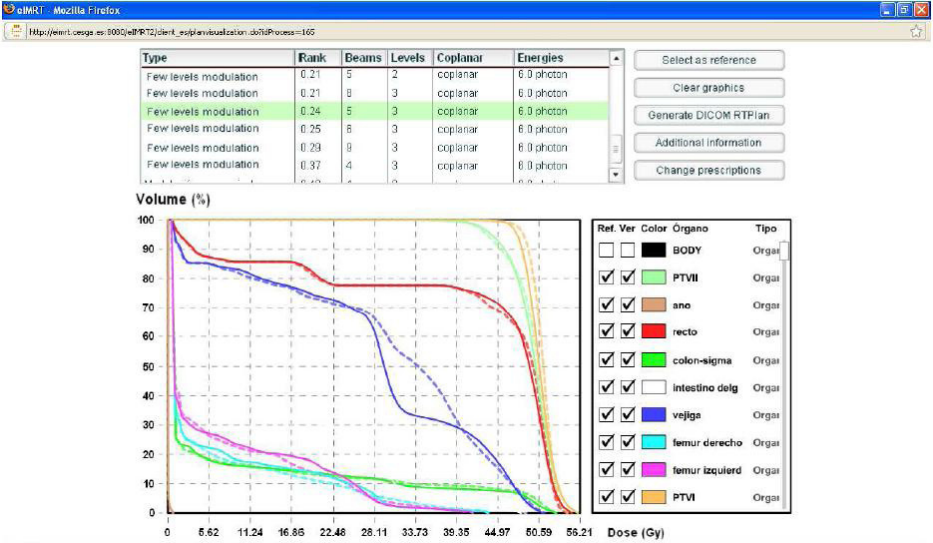
Flash tool for the review of treatment optimization results. The plans can be compared against a certain reference, retrieved in DICOM format or recalculated after changing the current prescriptions. Dashed lines correspond to a 9‐beam IMRT solution, while continuous lines are the DHVs histograms for a 5‐beam and three‐level discretization pseudo‐IMRT comparative solution.

The review of treatment optimization results is performed by comparing dose volume histograms of the calculated plans. Figure 7 shows the flash tool for this purpose. All the proposed plans are presented in a list, grouped by their types, and ranked according to their objective function value. Other information in this list includes the number of beams and levels, and whether the plan is coplanar or is of a single treatment modality.

The comparison among treatment plans is performed by selecting one of them as a reference. The DVH curves of the selected plan are plotted as dashed lines, and they are highlighted in red in the list. In the example in Fig. 7 for a prostate treatment, the best IMRT plan for nine beam incidences is selected as a reference. Then, by selecting any other plan from the list, their DVH curves are superimposed as solid lines. In this case, the advantage of employing 9 beams is shown by comparing it with a pseudo‐IMRT plan with 5 beams in which the fluence is discretized in three levels. One of the advantages of this pool solution strategy to the user is the ability of quickly exploring many alternative plans. In the example, it is clear than the two selected treatments have similar quality in terms of their Dose‐Volume histograms, while the pseudo‐IMRT represents a simpler solution from the treatment‐delivery point of view.

In the case that one of the treatments fulfills the user requirements, its associated DICOM RT Plan file can be directly downloaded from this page. Otherwise, the user can change the constraints or weights of the different organs with the single limitation of respecting their type (“treat” or “spare”). For a successful optimization, the weight of each organ is less relevant than an accurate prescription of constraints for PTVs within other PTVs and OAR/PTV overlapping regions.

## IV. DISCUSSION & CONCLUSIONS

The objective of the eIMRT project was the development of an open remote distributed computing platform in order to provide hospitals with computing resources that allow for an increase of the quality of radiation treatments. Although it is not ready to become a mass‐production environment in its current form, it represents an excellent example of the synergies that may be established among high‐performance computing, Internet technologies, and radiotherapy applications. This platform represents an evolution from desktop‐based to service‐based solutions for radiotherapy. As no prior experience in Monte Carlo or optimization is required to use the platform, many treatment sites may enjoy eIMRT services.

Although the user interfaces are flexible, simple, and user‐friendly, eIMRT algorithms are rather complex and include many improvements over those in conventional TPS. Again, we must emphasize that the open eIMRT architecture easily admits new algorithms as modules. The possibility of handling many users is a new feature, as compared to other in‐house Monte Carlo treatment verification solutions. An automated accelerator commissioning scheme that ensures machine reproducibility is combined with a rigorous QA procedure to obtain accurate treatment verifications.

Treatment optimization represents a very powerful tool that combines a highly accurate dose calculation algorithm with the evaluation of alternatives across a large solution space. The directions of incidence and the radiation intensity are optimized for different treatment delivery strategies covering CRT and IMRT, also providing other solutions with intermediate complexity.

By allowing the users to publish their treatments in a repository, eIMRT is also a platform for the sharing of knowledge. It is possible to take advantage of other experiences for speeding up the learning curve of IMRT implementation or increasing the quality of the procedures that are already in use. This feature also represents an excellent opportunity to explore the possibility of establishing standards for certain kind of treatments. This means that the sharing of treatment strategies for some type of disease may lead to the spread of uniformity in IMRT treatments.

The possibilities of this application are ample because of its flexibility. Treatment verification may serve both to recalculate certain treatments and to simulate simple beam arrangements in order to check the accuracy of TPS software in inhomogeneous media. Treatment optimization may serve to explore new treatment possibilities, to refine routine procedures, or to design custom solutions for particularly difficult cases.

This software is an open source platform that will be distributed through *gforge* (http://gforge.cesga.es/projects/eIMRT) *in the near future.* In this way, it will be available to any user, and will profit from different contributions in order to improve or add functionalities to the eIMRT project. This platform offers advanced treatment planning strategies in an open environment that will contribute to reduce healthcare costs and enhance the quality of treatments previously based only on local computing resources.

## ACKNOWLEDGEMENTS

This work was financed by Xunta de Galicia of Spain through grant PGIDT05SIN00101CT and by the European Community through the BeInGrid project. The authors are indebted to Dr. S. Naqvi for providing his Monte Carlo C/S dose calculation code and to the Medical Physics group of the University of Sevilla for the helpful discussions on Monte Carlo treatment verification.
